# COVID-19 Shock, Financial Flexibility, and Hotels' Performance Nexus

**DOI:** 10.3389/fpubh.2022.792946

**Published:** 2022-04-18

**Authors:** XueHui Zhang, Bao-Guang Chang, Kun-Shan Wu

**Affiliations:** ^1^School of Economics and Management, Inner Mongolia University of Technology, Huhhot, China; ^2^Department of Accounting, Tamkang University, New Taipei City, Taiwan; ^3^Department of Business Administration, Tamkang University, New Taipei City, Taiwan

**Keywords:** COVID-19, financial flexibility, firm performance, hotel industry, quantile regression

## Abstract

This study investigates the nexus of coronavirus disease 2019 (COVID-19) shock, financial flexibility (FF), and firm performance (FP) in Taiwan listed hotel firms. Quantile regression (QR) methods were used to analyze the data from Taiwan Stock Exchange listed hotel firms between 2020 Q1 and 2021 Q2. The results evidence that there is an inversed U-shaped linkage between FF and FP for the hotel industry. Additionally, FF has an inverted U-shaped effect on FP for the asset-light hotel firms for all quantiles except the 50th quantile. In addition, FF also has an inverted U-shaped impact on FP for the asset-heavy hotel firms in the 10th and 90th quantiles. A significant finding in this study is that there is a concave non-linear relationship between FF and FP, consistent with the law of diminishing marginal return. That is, with an increase in FF, the FP is on the rise; when FF exceeds the inflection point level, the FP begins to decline. Thus, a firm must ensure that the FF strategy it adopts must be the most efficient and effective, i.e., it must bring the trade-off between costs and benefits. The empirical results highlight the need for the hotel industry of Taiwan to take the rolling adjustment and optimization of FF after the COVID-19 pandemic for long-term sustainability.

## Introduction

The coronavirus disease 2019 (COVID-19) had spread across the world at an unprecedented rate, which has caught governments off guard and unprepared for such an impactful pandemic. Strict regulations, such as state of emergencies, stay-at-home orders, and border closings, have contributed to serious economic consequences (1). The impact on enterprises within the hospitality and travel industries, in particular the hotel industry, has been dramatic (2, 3). The decline in family and business travel has caused a direct impact on the performance of the entire hotel industry during the COVID-19 shock (4). According to the latest IBISWorld Industry Report for Hotels and Motels in the United States, the hotel and motel industry has been one of the industries severely hit by the COVID-19 pandemic, and industry revenues are expected to fall 45.7% alone in 2020 to the record lows of $107.7 billion (5). The hotel industry of Taiwan has been severely affected, during the first half of 2020, the industry revenue of hotels decreased by more than NT $12.863 billion from the same period last year, a significant drop of 43.39% from the same period last year (6).

Financial flexibility (FF) is the capability of a financial enterprise to obtain and restructure the required finance for minimum cost (7) and can help firms respond to the market changes affecting investment, performance, and business growth. Even in a crisis, companies with adequate FF have greater cash reserves to economically raise capital to fund new growth opportunities and further improve performance (8). Recently, enterprises worldwide have looked to increase their FF to avoid uncertainty and seize growth opportunities (9, 10).

Several researchers have explored the challenges faced by the hotel industry and its corporate response to the COVID-19 shock (11–14); nevertheless, there is no empirical research that evaluates the effect of the COVID-19 epidemic on the firm performance (FP) of the hotel industry. This study not only offers additional empirical evidence on the effect of the COVID-19 epidemic but also provides a deeper understanding of how FF affects FP amid a COVID-19 crisis. The literature concerning the relationship between FF and FP is inconclusive. There are numerous studies that indicate a positive FF-FP relationship (15–18), conversely, some articles argue that high FF leads to overinvestment (19).

Recently, in the research of Fahlenbrach et al. (20), firms with high FF experienced a stock price drop lower by 26% than those with low FF accounting for a firm's industry. This effect was greater in industries that were more severely affected by the COVID-19 shock. Ramelli and Wagner (21) have shown that firms with FF experienced greater stock performance in the initial stages of the COVID-19 epidemic. In addition, the scholar (22) confirmed that FF has a positive impact on FP for the Taiwanese manufacturing industry during the COVID-19 shock. Some studies have focused on the nonlinear concave (inverted U-shaped) relationship between FF and FP (23, 24). The inverted U-shaped curve demonstrates that initially FP rises as FF increases; however, on reaching the FF threshold, FP declines as FF increases. Until now, there is no consensus as to whether FF increases or decreases FP during the COVID-19 shock. Prompted by the ongoing debate, a primary enquiry of this research is whether FF impacts FP and more specifically, how it effects FP. In addition, we compare the FF-FP nexus during the pre- and post-COVID periods.

This research contributes to the extant literature and helps in bridging this gap in previous studies by exploring the impact of FF on FP for Taiwan listed hotel firms, considering a non-linear association. By utilizing the quantile regression (QR) method, this study addresses the tail information of FP (proxies by return on equity [ROE]) and identifies how FF affects different FF quantiles. Moreover, this article investigates whether the FF-FP nexus varies with different hotel industries' operational characteristics (asset-heavy and asset-light business models). These contributions highlight the importance of this study.

This study has several significant findings. The first analysis shows a convex (inverted U-shaped) FF-FP nexus in Taiwan hotel firms during 2020 Q1–2021 Q2. QR approach displays a concave FF-FP nexus in all ROE quantiles except the 25th quantile. That is, with an increase in FF, the FP is on the rise, however, FP is beneficial only up to a threshold point, after which there are diminishing marginal returns to FP. In other words, the hotel firm must ensure that the FF strategy it adopts must be the most efficient and effective, i.e., it must bring the trade-off between costs and benefits. The second analysis shows the linkage between FF and FP following different patterns for the asset-heavy business model (AHBM) and asset-light business model (ALBM) hotel firms. For the AHBM firms, the QR approach evidence a concave (inverted U-shaped) FF-FP nexus in the 10th and 90th quantiles. Similarly, the QR approach shows a concave FF-EP nexus in all ROE quantiles except the 50th quantile, in the ALBM hotel firms. The third analysis supports the inverted U-shaped nexus between FF and FP during the COVID-19 while no relationship exists before the COVID-19. This finding may indicate some structural changes in the FF of the hotel industry between the two periods due to the COVID-19 pandemic.

The rest of the analysis is divided into five parts after the introduction. The research history and current results on the effects of FF on FP are clarified in the literature review section. The research methodology segment describes the sample and data collection process, research model, and methods. We present the details of the empirical results in the fourth segment. The discussion of findings and their implication is described in the fifth segment, and finally, Section 6 concludes the study.

## Literature Review and Hypothesis Development

### COVID-19 Shock in the Hotel Industry

The tourism and hospitality industry is especially affected by health emergencies (25–27). For example, the hotel industry is one of the most affected as domestic and foreign travel has been restricted to prevent the spread of COVID-19. The sensitivity of the hotel industry to external shocks compared to other service industries, combined with its higher fixed assets, higher fixed costs, and higher leverage structures, makes the hotel industry more vulnerable to the COVID-19 pandemic (28, 29).

Recent research has found that the financial strength of a company is becoming particularly important: stock market prices are less affected by the crisis in firms with more cash holdings, lower leverage, and more profits (21, 30, 31). Recent papers have focused on the impact of the pandemic on the stock prices and value creation of hotel companies. Wu et al. (32) adopted the event research method to explore the impact of COVID-19 on the stock price fluctuations of China's tourism industry. The results show that the COVID-19 crisis has had a negative impact on the hospitality and tourism sector stocks of China.

### Effect of Financial Flexibility on Firm Performance

The resource-based view argues that enterprises with idle or surplus resources can use these resources to obtain external opportunities and promote enterprise growth (33). In addition, the tradeoff theory (34) states that when firms experience financial difficulties, sufficient cash reserves help reduce risks (24). The agency theory (35, 36), however, predicts that when a firm has extra cash, managers may waste it or invest in detrimental projects. Additionally, an excessive FF will lead to excessive idle cash, making the profitability of corporate cash relatively weak. Conversely, however, the low debt and low leverage have no incentive effect and will reduce FP (24).

Despite the extensive previous literature on the FF-FP nexus, there are still competing views. Chun and Yanbo (15) examined whether investment scale or efficiency guides the FF-FP relationship for companies listed on the Shanghai and Shenzhen stock exchanges (SSSE) and showed that FF significantly positively impacts FP. Arslan-Ayaydin et al. (8), from 1994 to 2009, assessed the impact of FF on FP of East Asian firms and indicated that corporations with optimum FF perform better in a financial crisis. Al-Slehat (16), from 2010 to 2017, explored the impact of FF on the FP of service industries in Jordan and found it had a positive influence.

Mehmood et al. (37) reported that FF had a positive influence on FP for the Pakistan Stock Exchange listed enterprises, from 1992 to 2014. The same positive relationship for Pakistani enterprises was also reported by Ali and Siddiqui (17), from 2009 to 2018. Recent research had shown that firms with FF experienced greater stock performance in the initial stages of the COVID-19 epidemic (20, 21). In addition, the scholar verified that FF has a positive impact on FP for the Taiwanese manufacturing industry during the COVID-19 shock (22). Contrastingly, some studies suggest that high FF leads to overinvestment from an agency costs perspective (19).

Recently, mixed results on the impact of FF on FP have led to doubt surrounding the linear relationship between the two variables, thus instigating the adoption of nonlinear models. Some studies concentrating on the nonlinear FF-FP relationship evidence that it has a concave (inverted U-shaped) pattern. For example, Yi (23) explored the impact of FF on the FP of SSSE listed manufacturing firms, from 2011 to 2017, and found the relationship to be concave. Gu and Yuan (24), from 2015 to 2018, investigated the associations among internal control, FF, and FP of SSSE listed Chinese information technology companies and confirmed that FF has a concave effect on FP. Chang and Wu (38) applied QR to analyze the effect of FF on FP for the Taiwan Stock Exchange (TSE) listed semiconductor firms, from Q1 2020 to Q1 2021, and evidenced a concave FF-FP nexus.

The aforementioned studies argue that improvement to the level of FF assists enterprises to achieve optimal performance. When FF exceeds the threshold (inflection point) level, the level of FF has a negative impact on FP. Consequently, the present study allows for the presence of both the positive and negative effects of FF levels on the performance of hotel firms by applying a concave function, as in Yi (23), Gu and Yuan (24), and Cang and Wu (38). Therefore, the first hypothesis is proposed:

**H1**. The effect of FF on FP is non-linear and assumes an inverted U-shaped form.

Recently, major hotel companies have increasingly reduced their ownership of hotel assets in the favor of ALBM (39). Some studies, from the dynamic capability's perspective, consider that ALBM is one of the unique dynamic capabilities of hotel enterprises, which can modify and update existing resources as per the environmental changes, so as to gain advantages over competitors and achieve excellent FP (40–42). In addition, some studies identified both positive and negative effects on ALBM (AHBM) on FP, nevertheless, the results of these articles were contradictory and inconclusive. Sohn et al. (43) verified a positive correlation between the ALBM, operating profitability, and enterprise value, indicating that ALBM improved FP. Seo et al. (44) also indicated a positive relationship between ALBM and FP for the United States loading firms. Conversely, Blal and Bianchi (45) found that the ALBM model had no impact on FP, e.g., share returns, Earnings Before Interest, Taxes, Depreciation, and Amortization (EBIDTA), and ROE, of the six leading U.S. corporations over a 16-year period. On the other hand, Low et al. (46) found that AHBM hotel companies were preferred over ALBM hotel companies. Consequently, based on the above discussion, the second and third hypotheses are as follows:

**H2**. There is a significant positive relationship between FF and FP in AHBM hotel firms.
**H3**. There is a significant positive relationship between FF and FP in ALBM hotel firms.

## Research Methodology

### Sample and Data

The sample included all 20 publicly traded hotel firms listed on the TSE. The sample hotel companies provided financial data from Q1 2020 to Q2 2021. Taiwan Economic Journal (TEJ) database provides the financial and accounting data of firms to measure FP. The quarter values of one firm were missing from Q4 2020 due to the recompilation of their financial reports, as a result, 119 quarterly sample observations were available.

### Variables

#### Dependent Variable

ROE commonly appears in the empirical board literature as holistic measures of FP (47, 48). To measure the performance and the ability to generate profits, the ROE of different hotel firms was compared. ROE is operationalized as the net income divided by shareholder equity.

#### Independent Variable

FF is the independent variable. Since there is no unified standard method for FF measurement, this article refers to the finding of other authors (17, 22), where FF is computed as FF = Cash flexibility + Debt flexibility.

#### Control Variables

The following control variables were selected as previous studies found they have an effect on FP: growth rate of revenue (REVG) is referred to as the quarter-over-quarter percentage increase in revenue; current ratio (RDG) is calculated as current assets/current liability; net profit growth rate before taxes (BNIG) is measured by “net profit before taxes for the current period minus net profit before tax of the previous period divided by net profit before tax of the previous period”; the growth rate of owner's equity (OEG) is captured as the percentage change in owner's equity over the prior period (49, 50); average collection days (ARD) (51, 52); and SIZE (firm size (SIZE) is measured by the natural logarithm of total assets (48, 53).

### Research Model

In hospitality and tourism research, it is customary to use the ordinary least square (OLS) regression to test the hypotheses (54), as it captures the relationships at the mean. Nonetheless, focusing on central influences may lead to the underestimation or overestimation of correlation coefficients or failure to identify important associations, which may result in false positives and ignoring information at the tail of the distribution (55). As quantile regression (QR) allows for a full range of conditional quantile functions (55) and is more robust, providing more efficient estimations, besides, QR can bring an additional research advantage in tourism studies (54, 56). Hence, this research utilizes Koenker and Bassett's (57) QR model as follows.


(1) $$\begin{array}{l} Q_{\theta }\left(ROE_{it}|X_{it}\right)=\beta_{0\theta }+\beta_{1\theta }FF_{it}+\beta_{2\theta }FF2_{it}+\beta_{3\theta }CON_{it}\\ \;+\varepsilon_{\theta it} \end{array}$$

where *Q*_θ_(*ROE*_*it*_|*X*_*it*_) is the θ-th QR function. *ROE*_*it*_ is the FP of *i* firm in *t* quarter; *FF*_*it*_ is the FF of *i* firm in *t* quarter; *FF*2_*it*_

is the square of FF for *i* firm in *t* quarter; *CON*_*it*_ is referred to as the control variables in the model including quarter and industry level controls; ε_θ*it*_ represents error terms for firm *i* at quarter *t* at the θ-th quantile. The aforementioned QR model explores the nonlinear FF-EP nexus within financial quarters, after heteroscedasticity adjustment with a cluster at the firm level.

Furthermore, as described by the authors (58–60), it is best to use bootstrapping as an effective robust resampling technique to obtain healthy estimated results when the sample in the empirical model using quantile regression is small. In a recent study, academics have used QR as an available bootstrap method in statistical analysis software, such as STATA (61). These are standard methods for estimating the asymptotic covariance matrix of coefficients (62). In this study, we employed the QR approach to examine whether the determinants' effects are distinguishable across the quantiles in the conditional distribution of the dependent variable. In our estimations, 200 bootstrap replications are set to guarantee a small sample variability of the covariance matrix.^1^

## Results

### Descriptive Statistics

Table 1 shows the descriptive statistics of the variables used in this study. Before the COVID-19, the mean of FF for the TSE listed hotel firms is 54.804%. The mean (median) of ROE is 0.966% (0.57%), the minimum is −63.83%, and the maximum is 173.46%. During the COVID-19, the average level of FF for the hotel firms is 53.203%. For ROE, the mean value is −17.662%, the median is −0.63%, the minimum is −714.92%, and the maximum is 24.69%. The mean value of ROE is lower than the median, and there is a wide range between the minimum and maximum values. The skewness value is −6.198 and the kurtosis value is 42.315, which illustrates that the distribution of ROE is skewed and heavily left-tailed. The normality test on ROE verifies that its Jacque-Bera statistic (=83,000, *p* < 0.001) rejects the hypothesis of ROE normally distributed.

**Table 1:** Summary statistics.

**Variables**	**ROE**	**FF**	**REVG**	**RDG**	**BNIG**	**OEG**	**ARD**	**SIZE**
**Before COVID-19 period: Q3 2018~** **Q4 2019**								
Mean	0.966	54.804	4.225	2.015	−43.993	19.308	24.309	15.072
St. deviation	18.374	27.488	78.863	3.704	876.811	196.421	54.569	0.918
Min	−63.83	2.825	−285.66	0.149	−8196.67	−76.16	0.02	13.192
Max	173.46	99.38	697.83	20.761	3598.42	2112.92	429.44	16.59
10th percentile	−5.22	16.477	−40.135	0.285	−182.375	−21.255	4.425	13.884
25th percentile	−1.255	32.085	−8.37	0.437	−38.95	−7.12	7.845	14.248
50th percentile	0.57	53.996	1.455	0.838	5.905	0.825	11.31	14.995
75th percentile	1.905	81.414	7.655	1.545	70.9	6.635	15.145	15.963
90th percentile	6.05	91.936	35.635	4.136	214.1	19.47	23.57	16.291
Skewness	6.538	−0.052	5.468	3.524	−6.318	10.253	5.035	−0.086
Kurtosis	67.494	1.819	53.043	15.467	66.294	109.443	31.702	1.965
Sample sizes	120	120	120	120	120	120	120	120
Jarque-Bera test = 842								
**During COVID-19 period: Q1 2020** **~** **Q2 2021**								
Mean	−17.662	53.203	−13.043	2.041	−2,513.254	10.69	34.659	15.085
St. deviation	90.378	29.308	45.374	3.824	32,438.193	119.124	100.608	0.955
Min	−714.92	2.029	−136.15	0.046	−284984	−88.67	0	13.279
Max	24.69	103.369	179.92	21.159	1,02,000	1,073.57	856.13	16.578
10th percentile	−14.02	14.453	−61.7	0.245	−1,440.41	−30.05	1.01	13.816
25th percentile	−5.46	26.675	−43.3	0.427	−142.03	−15.41	6.52	14.196
50th percentile	−0.63	53.747	−17.23	0.871	−29.51	−3.19	10.51	15.143
75th percentile	0.84	83.1	8.28	1.524	82.77	2.84	17.45	16.059
90th percentile	4.45	91.654	44.13	4.947	253.28	10.61	63.96	16.323
Skewness	−6.198	0.039	1.07	3.479	−6.076	7.4	5.96	−0.054
Kurtosis	42.315	1.599	6.167	15.029	55.07	61.435	43.391	1.793
Sample sizes	119	119	119	119	119	119	119	119
Jarque-Bera test = 83,000								

### Comparisons of Before-COVID-19 and During-COVID-19

Further analyses of before-COVID-19 and during-COVID-19, however, reveal some interesting findings: OLS regression reveals the inverted U-shaped nexus between FF and FP during the COVID-19 while no relationship exists before the COVID-19, as shown in Table 2. In addition, this study uses the seemingly unrelated estimation (SUE) (64) to adjust standard errors (SEs) simultaneously to compare the coefficients from the OLS estimated results before COVID-19 and during COVID-19. To be specific, the gaps between any two coefficients were tested to determine if gaps were equal to zero or not by using the Hausman test (65). From Table 2, it is possible that the effect of independent and control variables differed across periods.

**Table 2:** Regression results: before-coronavirus disease 2019 (COVID-19) vs. during-COVID-19.

**Variables**	**ROE**		**χ^2^**
	**Before-COVID**	**During-COVID**	
FF	−0.5099	7.1832***	9.66***
	(0.7415)	(2.5035)	
FF2	0.0039	−0.0550***	8.91***
	(0.0056)	(0.0200)	
REVG	−0.0051	0.3115**	4.78**
	(0.0119)	(0.1458)	
RDG	0.1598	−3.5975**	6.82***
	(0.3693)	(1.6081)	
BNIG	0.0041**	−0.0001	9.25***
	(0.0017)	(0.0001)	
OEG	−0.0384***	0.0902**	12.32***
	(0.0112)	(0.0360)	
ARD	0.0011	−0.0564	1.15
	(0.0186)	(0.0503)	
SIZE	−0.5351	32.8389***	8.13***
	(2.4878)	(12.2472)	
Constant	21.8171	−684.6893***	9.03***
	(55.6969)	(249.9651)	
Sample size	120	119	
Quarter fixed effect	Yes	Yes	
R-squared	0.2305	0.4328	
Breusch-Pagan test of independence: χ^2^ (18) = 337.38, *p-*value =0.0000 < 0.01			

*Standard errors (SEs) are shown in parentheses*.

** *p < 0.05*.

*** *p < 0.01*.

Furthermore, the findings may indicate some structural changes in the FF of the hotel industry between the two periods due to the COVID-19 pandemic. Perhaps the ALBM employed by many hospitality firms (e.g., franchising strategy), further maturing in 2020, may have contributed to some of the discrepancies seen in this study. Thereafter, this study only concerns the effect of FF on EP amid the COVID-19 epidemic period.

### Empirical Results

First, whether multi-collinearity exists among the independent variables was investigated. Table 3 contains the results of the variance inflation factor (VIF) on the independent variable with a mean VIF of 1.20. The highest value is 1.56, which is far below the cut-off value of 10, suggested by Hair et al. (66). Therefore, no multicollinearity problem is suspected.

**Table 3:** Correlation matrix.

**Variables**	**(1)**	**(2)**	**(3)**	**(4)**	**(5)**	**(6)**	**(7)**
(1) FF	1						
(2) REVG	0.204**	1					
(3) RDG	0.463***	0.049	1				
(4) BNIG	0.162*	0.122	0.039	1			
(5) OEG	−0.019	−0.029	−0.015	0.050	1		
(6) ARD	−0.109	0.122	−0.088	0.011	0.080	1	
(7) SIZE	−0.115	−0.078	0.315***	−0.082	−0.199**	−0.231**	1
VIF	—	1.08	1.56	1.04	1.06	1.11	1.37

* *p < 0.1*.

** *p < 0.05*.

*** *p < 0.01*.

Next, Table 4 exhibits the results of the OLS and QR analysis for the full hotel industry. OLS regression estimation results reveal that the coefficients of FF and FF2 are positive and negative (*p* < 0.01), respectively, and both are statistically significant, indicating the nonlinear (inverted U-shaped) FF-FP nexus. Additionally, the QR approach reveals that the coefficients of FF and FF2 are positive and negative (*p* < 0.01), respectively, and both are statistically significant in all quantiles except the 25th quantile. This shows that the nexus between FF and FP is a nonlinear inverted *U*-shaped form in all quantiles, except the 25th quantile (Table 4). Thus, H1 is supported.

**Table 4:** Regression results during the COVID-19 pandemic period.

**Variables**	**OLS**	**Lower quantiles**		**Median**	**Upper quantiles**	
		**10th quantile**	**25th quantile**	**50th quantile**	**75th quantile**	**90th quantile**
FF	7.1832***	11.8027***	0.9535	0.5303***	0.3306***	0.4166**
	(2.5035)	(4.2314)	(1.8671)	(0.0991)	(0.1045)	(0.1657)
FF2	−0.0550***	−0.0881**	−0.0070	−0.0037***	−0.0024**	−0.0034**
	(0.0200)	(0.0397)	(0.0175)	(0.0009)	(0.0010)	(0.0016)
REVG	0.3115**	−0.0222	0.0319	0.0349**	0.0377**	0.0678***
	(0.1458)	(0.6538)	(0.2885)	(0.0153)	(0.0162)	(0.0256)
RDG	−3.5975**	0.6719	−0.1139	−0.0574	−0.0056	−0.0795
	(1.6081)	(8.3158)	(3.6693)	(0.1947)	(0.2055)	(0.3257)
BNIG	−0.0001	−0.0005	0.0000	0.0000	0.0000	0.0000
	(0.0001)	(0.0008)	(0.0003)	(0.0000)	(0.0000)	(0.0000)
OEG	0.0902**	0.1240	0.0071	0.0017	−0.0033	−0.0094
	(0.0360)	(0.2235)	(0.0986)	(0.0052)	(0.0055)	(0.0088)
ARD	−0.0564	0.0131	−0.0029	−0.0094	−0.0080	−0.0109
	(0.0503)	(0.2668)	(0.1177)	(0.0062)	(0.0066)	(0.0104)
SIZE	32.8389***	18.5571	3.0050	1.0232	−0.2665	−0.8683
	(12.2472)	(30.6959)	(13.5443)	(0.7188)	(0.7584)	(1.2022)
Constant	−684.6893***	−673.9087	−78.9195	−35.7022***	−6.6004	5.1965
	(249.9651)	(475.3999)	(209.7664)	(11.1316)	(11.7454)	(18.6185)
Sample size	119	119	119	119	119	119
Quarter Fixed Effect	Yes	Yes	Yes	Yes	Yes	Yes
R-squared/ Pseudo R^2^	0.4328	0.2922	0.1076	0.0904	0.0792	0.1239

*SEs are shown in parentheses*.

** *p < 0.05*.

*** *p < 0.01*.

Regarding the control variables, OLS estimation results show that REVG significantly positively impacts FP, whereas QR results evidence that the positive effect of REVG is predominantly in the 50th, 75th, and 90th quantiles. The OLS estimation results of RDG verify that there is a significant negative effect; nevertheless, this is not apparent in the QR estimation results. BNIG and ARD are neither significant in the OLS nor QR estimation results. OLS estimation results expose that OEG has a positive effect; however, this is not apparent in the QR results. OLS analysis reveals that the SIZE has a positive effect; however, this is not apparent in the QR results (Table 4).

### Inter-quantile Difference

Results confirmed that the impact of FF (including its components) on FP is heterogeneous across the ROE distributions. Inter-quantile regression was employed to test whether the slope of the entire quantile is equal to verify that the difference is statistically significant (57). The F statistic formula was also used to test the equality of the coefficient across various quantile pairings. Table 5 shows the results of the *F*-test and the corresponding values of *p* after examining the uniformity of the coefficient only between the upper (90th) and lower quantiles (10th), using the bootstrap process with 200 replications. Table 5 presents the inter-quantile results for ROE. For the whole hotel, there are statistically significant differences in the parameter estimates of FF and FF2 for the symmetrical quantiles [quantile (90/10)].

**Table 5:** Inter-quantile regression results.

**Q (90/10)**	
FF	F-statistics	4.57
	Significance	0.0348**
FF2	F-statistics	4.63
	Significance	0.0338**
REVG	F-statistics	0.13
	Significance	0.7169
RDG	F-statistics	0.43
	Significance	0.5125
BNIG	F-statistics	0.28
	Significance	0.5963
OEG	F-statistics	0.63
	Significance	0.4310
ARD	F-statistics	0.00
	Significance	0.9624
SIZE	F-statistics	2.99
	Significance	0.0865*

*Q(90/10) = 90th quantine(y)−10th quantine(y)*.

* *p < 0.1*.

** *p < 0.05*.

In addition, the business model of the hotel generally distinguishes between the AHBM and ALBM. Thus, this paper divides the full hotel industry into the AHBM and ALBM hotel firms. The asset-light business model of hotel firms was measured through the ratio of the fixed assets (property, plant and equipment, PPE) to total assets ratio (67) quarter-by-quarter. Consequently, the subsample of 65 AHBM firms was above the average fixed assets to total assets ratio, and the subsample of 54 ALBM firms was below the average fixed assets to total assets ratio. Tables 6, 7 show the estimation results for these subsamples.

**Table 6:** Quantile regression results of the AHBM hotel firms.

**Variables**	**OLS**		**Lower quantiles**		**Median**	**Upper quantiles**	
			**10th quantile**	**25th quantile**	**50th quantile**	**75th quantile**	**90th quantile**
FF	0.0769	0.3493**	0.5072***	0.3333	0.1782	0.0786	0.3135**
	(0.0711)	(0.1327)	(0.1412)	(0.2106)	(0.1380)	(0.2106)	(0.1546)
FF2		−0.2522**	−0.0030**	−0.0019	−0.0012	−0.0009	−0.0035**
		(0.1075)	(0.0013)	(0.0019)	(0.0012)	(0.0019)	(0.0014)
REVG	0.0599***	0.0698***	0.0525**	0.0620	0.0251	0.0224	0.0075
	(0.0217)	(0.0247)	(0.0249)	(0.0372)	(0.0244)	(0.0372)	(0.0273)
RDG	0.2414	0.8008	−0.4501	0.8372	1.5531	2.1073	4.2763***
	(1.6719)	(1.6713)	(1.4017)	(2.0902)	(1.3698)	(2.0905)	(1.5344)
BNIG	−0.0000	−0.0000	0.0000	0.0000	0.0000	0.0000	0.0000
	(0.0000)	(0.0000)	(0.0001)	(0.0001)	(0.0001)	(0.0001)	(0.0001)
OEG	−0.0173	−0.0363	−0.0732**	0.0032	−0.0098	−0.0261	−0.0422
	(0.0320)	(0.0314)	(0.0282)	(0.0420)	(0.0275)	(0.0420)	(0.0309)
ARD	−0.1893	−0.2203*	−0.2812*	−0.1574	−0.2013	−0.2806	−0.5356***
	(0.1295)	(0.1283)	(0.1498)	(0.2233)	(0.1464)	(0.2234)	(0.1639)
SIZE	−0.3954	−0.5108	0.8867	−0.1843	−1.5762	−2.1118	−3.9646***
	(1.6899)	(1.6614)	(0.9952)	(1.4839)	(0.9725)	(1.4841)	(1.0894)
Constant	2.9779	−0.1821	−32.7614**	−13.2931	14.9431	29.9414	57.2015***
	(28.4147)	(27.9549)	(16.0164)	(23.8828)	(15.6518)	(23.8859)	(17.5326)
Sample size	65	65	65	65	65	65	65
Quarter Fixed Effect	Yes	Yes	Yes	Yes	Yes	Yes	Yes
R-squared/ Pseudo R^2^	0.4903	0.5225	0.5624	0.4664	0.3848	0.3702	0.4340

*SEs are shown in parentheses*.

* *p < 0.1*.

** *p < 0.05*.

*** *p < 0.01*.

**Table 7:** Quantile regression results of the ALBM hotel firms.

**Variables**	**OLS**		**Lower quantiles**		**Median**	**Upper quantiles**	
			**10th quantile**	**25th quantile**	**50th quantile**	**75th quantile**	**90th quantile**
FF	2.8727***	11.6715***	17.4857***	11.6755***	1.1877	0.9359***	0.8519***
	(1.0243)	(3.1459)	(2.6735)	(3.8234)	(1.5767)	(0.1423)	(0.0219)
FF2		−0.1066***	−0.1303***	−0.0887*	−0.0094	−0.0082***	−0.0068***
		(0.0292)	(0.0307)	(0.0439)	(0.0181)	(0.0016)	(0.0003)
REVG	0.1466	−0.0106	−0.1202	−0.0878	0.0012	0.0173	0.0186***
	(0.2848)	(0.1860)	(0.3767)	(0.5387)	(0.2221)	(0.0200)	(0.0031)
RDG	−11.0053**	2.6470	1.0378	0.0394	−0.0539	0.2000	−0.0575
	(4.3514)	(2.1619)	(5.8956)	(8.4314)	(3.4769)	(0.3137)	(0.0483)
BNIG	−0.0003	−0.0001	−0.0007*	−0.0004	−0.0000	0.0000	0.0000***
	(0.0002)	(0.0002)	(0.0004)	(0.0005)	(0.0002)	(0.0000)	(0.0000)
OEG	0.1912**	0.1221**	0.1863*	0.1231	0.0091	0.0026	0.0031***
	(0.0729)	(0.0516)	(0.1050)	(0.1502)	(0.0619)	(0.0056)	(0.0009)
ARD	0.1914	0.0714	0.0353	0.0361	0.0076	0.0046	0.0161***
	(0.1186)	(0.0797)	(0.1313)	(0.1878)	(0.0774)	(0.0070)	(0.0011)
SIZE	78.1766***	68.1093***	28.1518	31.4854	6.4851	4.6943***	5.0453***
	(26.6061)	(21.1040)	(20.2077)	(28.8994)	(11.9174)	(1.0754)	(0.1656)
Constant	−1,344.20***	−1,327.69***	−997.27***	−843.27*	−134.30	−98.54***	−102.34***
	(446.3582)	(3.1459)	(2.6735)	(3.8234)	(1.5767)	(0.1423)	(0.0219)
Sample size	54	54	54	54	54	54	54
Quarter Fixed Effect	Yes	Yes	Yes	Yes	Yes	Yes	Yes
R-squared/ Pseudo R^2^	0.5165	0.6764	0.6672	0.3091	0.1112	0.1030	0.1531

*SEs are shown in parentheses*.

* *p < 0.1*.

** *p < 0.05*.

*** *p < 0.01*.

For the AHBM hotel companies, the OLS estimation results show that FF has not significantly positively influenced FP, which does not support H2 (Table 6). However, the QR approach reveals an inverted U-shaped FF-FP nexus in the lowest (10th) and the highest (90th) quantiles. For ALBM hotel firms, OLS regression reveals a significantly positive impact on FP, which does support H3 (Table 7). Furthermore, OLS verified that there is an inverted U-shaped (concave) FF-FP nexus and that QR had a concave impact on FP in all quantiles except the median quantile, again demonstrating that FF had a nonlinear concave effect on FP.

### Robustness Test

To perform a robustness test to check the consistency of the results, the model was estimated using different subsamples. This study utilizes firm size as a proxy for information asymmetry according to the current literature conventions. It is assumed that large enterprises have a low degree of information asymmetry and small enterprises have a high degree of information asymmetry and are treated as a separate sample (68). Thus, the subsample of 60 large firms was above the average value of nature logarithm of total assets, and the subsample of 59 small firms was below. Tables 8, 9 reveal the estimation results for these subsamples. The empirical results reveal that the FF and FF2 are positively and negatively significant, respectively, indicating a concave (inverted U-shaped) linkage between FF and FP, similar to the main consequences.

**Table 8:** Robustness test results of SIZE: large firms.

**Variables**	**OLS**	**Lower quantiles**		**Median**	**Upper quantiles**	
		**10th quantile**	**25th quantile**	**50th quantile**	**75th quantile**	**90th quantile**
FF	0.7314***	0.8048***	0.8630***	0.7909***	0.7383***	0.5840***
	(0.1262)	(0.1945)	(0.2462)	(0.1324)	(0.1381)	(0.0707)
FF2	−0.0061***	−0.0079***	−0.0075***	−0.0065***	−0.0065***	−0.0045***
	(0.0012)	(0.0019)	(0.0024)	(0.0013)	(0.0013)	(0.0007)
REVG	0.0644**	0.0408	0.0685	0.0361	0.0395	0.0988***
	(0.0308)	(0.0420)	(0.0532)	(0.0286)	(0.0298)	(0.0153)
RDG	−0.1064	0.1315	0.1016	−0.0588	0.0286	−0.2663***
	(0.1530)	(0.2650)	(0.3356)	(0.1804)	(0.1882)	(0.0963)
BNIG	0.0000	0.0000	0.0000	0.0000	0.0000**	0.0000***
	(0.0000)	(0.0000)	(0.0000)	(0.0000)	(0.0000)	(0.0000)
OEG	0.0211	0.0536	0.0233	−0.0034	−0.0272	−0.0255*
	(0.0190)	(0.0359)	(0.0454)	(0.0244)	(0.0255)	(0.0130)
ARD	−0.4369**	−0.9385***	−0.3379**	−0.3581***	−0.2581***	−0.2768***
	(0.1690)	(0.1318)	(0.1668)	(0.0897)	(0.0936)	(0.0479)
Constant	−9.0823**	−20.9386***	−25.7280***	−19.9127***	−15.4274***	−11.8064***
	(3.8437)	(4.2828)	(5.4229)	(2.9158)	(3.0411)	(1.5560)
Sample size	60	60	60	60	60	60
Quarter fixed effect	Yes	Yes	Yes	Yes	Yes	Yes
R-squared/ Pseudo R^2^	0.6450	0.5721	0.5188	0.4251	0.3774	0.4827

*SEs are shown in parentheses*.

* *p < 0.1*.

** *p < 0.05*.

*** *p < 0.01*.

**Table 9:** Robustness test results of SIZE: small firms.

**Variables**	**OLS**	**Lower quantiles**		**Median**	**Upper quantiles**	
		**10th quantile**	**25th quantile**	**50th quantile**	**75th quantile**	**90th quantile**
FF	12.8241***	18.7647***	11.5787***	0.4851	0.1824	−0.2873
	(3.6228)	(2.3187)	(3.5125)	(1.4931)	(0.2127)	(0.1642)
FF2	−0.0987***	−0.1365***	−0.0812**	−0.0028	−0.0009	0.0025
	(0.0291)	(0.0206)	(0.0312)	(0.0133)	(0.0019)	(0.0015)
REVG	0.4119*	0.1233	0.1484	0.0380	0.0160	0.0121
	(0.2109)	(0.3278)	(0.4966)	(0.2111)	(0.0301)	(0.0232)
RDG	−32.0693	−34.8215	−6.1910	1.2455	1.5060	1.5659
	(21.6539)	(36.3846)	(55.1181)	(23.4295)	(3.3379)	(2.5767)
BNIG	0.0004	0.0008	0.0003	0.0000	0.0000	−0.0000
	(0.0003)	(0.0009)	(0.0013)	(0.0006)	(0.0001)	(0.0001)
OEG	0.1010***	0.1895*	0.1237	0.0035	−0.0035	−0.0110
	(0.0364)	(0.0986)	(0.1494)	(0.0635)	(0.0090)	(0.0070)
ARD	−0.0818	0.0214	−0.0003	−0.0074	−0.0032	−0.0101
	(0.0665)	(0.1184)	(0.1794)	(0.0762)	(0.0109)	(0.0084)
Constant	−346.379***	−612.470***	−400.552***	−24.0144	−11.4133*	3.9073
	(97.6361)	(65.9778)	(99.9482)	(42.4858)	(6.0528)	(4.6725)
Sample size	59	59	59	59	59	59
Quarter fixed effect	Yes	Yes	Yes	Yes	Yes	Yes
R-squared/Pseudo R^2^	0.5971	0.6283	0.2319	0.0691	0.0758	0.0964

*SEs are shown in parentheses*.

* *p < 0.1*.

** *p < 0.05*.

*** *p < 0.01*.

## Discussion and Implications

This empirical investigation of the nonlinear FF-FP relationship uses OLS and QR analysis on data from the TSE listed hotel firms. The results elicit several findings regarding the relationship between FF and FP as follows.

**Figure 1:**
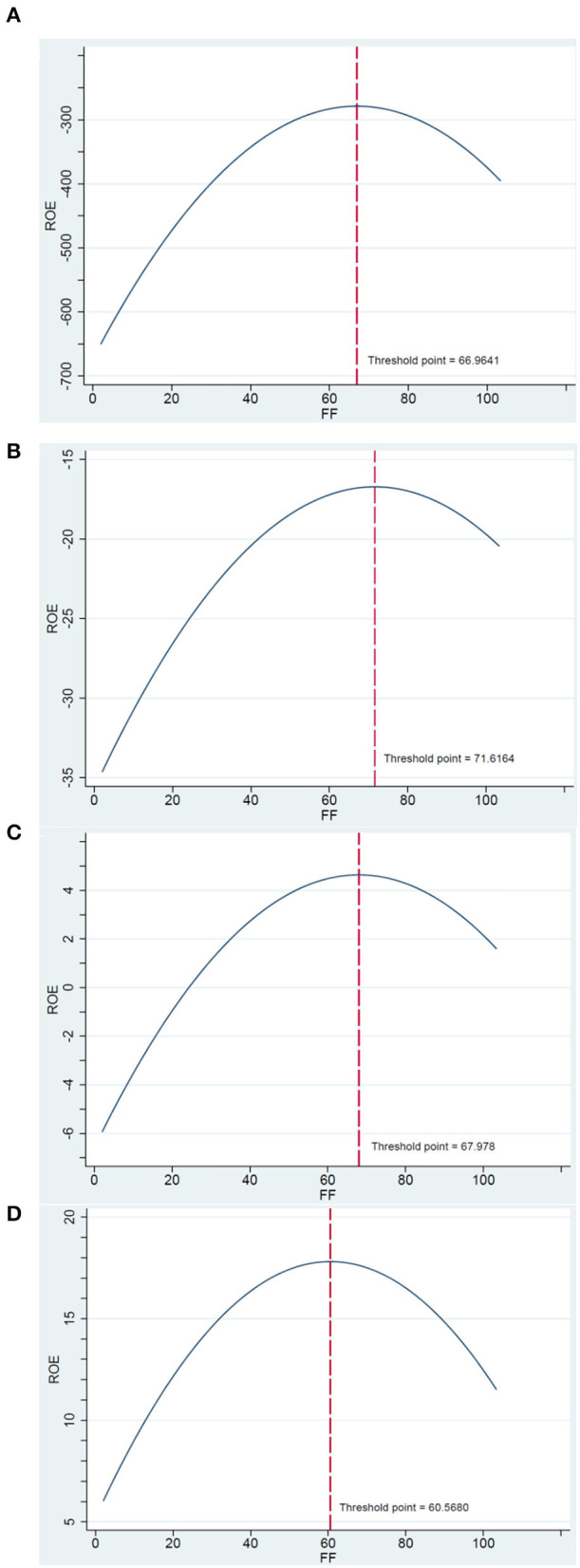
The inverted U–shaped financial flexibility (FF)-firm performance (FP) nexus for hotel companies in **(A)** 10th, **(B)** 50th, **(C)** 75th, and **(D)** 90th quantile.

This research verifies that the FF-FP nexus is a concave or an inverted U-shaped in all quantiles, except the 25th quantile. It indicates that the influence of FF on FP first increases and then decreases after reaching the optimal FF threshold. By solving the first derivative, the optimal FF values are 66.98, 71.66, 68.88, and 61.26% in the 10th, 50th, 75th, and 90th quantiles, respectively, as reported in Table 4 and shown in Figures 1A–D. Figures 1A–D indicates that FF can start enhancing the firm performance before the threshold point and reach the optimal firm performance at the threshold. Thus, the hotel firms should focus on the dynamic control and optimization of FF to obtain the maximum FP.

With regarding to AHBM hotel firms, the study evidenced a concave FF-FP nexus in the 10th and 90th quantiles. In addition, the optimal numbers of FF are 84.53 and 44.79% in the 10th and 90th quantile, respectively, as reported in Table 6. Similarly, for the ALBM hotel firms, the study also shows a concave FF-EP nexus in all ROE quantiles except the 50th quantile. In addition, the optimal numbers of FF are 67.1, 65.81, 57.07, and 62.67% in the 10th, 25th, 75th, and 90th quantiles, respectively, as reported in Table 7. Thus, either AHBM or ALBM hotel firms should focus on the dynamic control and optimization of FF to obtain the maximum FP.

To sum up, we find an inverted U-shaped relationship between FF and FP. Increases in FF improve FP. However, FP is beneficial only up to a point, after which there are diminishing marginal returns to FF. The inverted *U*-shaped FF-FP nexus is guided by a trade-off. Thus, a firm must have an efficient FF in place that balances the cost and benefits. More specifically, the hotel industry of Taiwan (includes AHBM and ALBM firms) set target FF based on the balance between the marginal benefits and costs of FF. These results confirm that the FF plays a significant role in the decision-making of Taiwanese hotel firms.

## Conclusion

This study adds to the literature by showing that the FF-FP nexus follows an inverted U-shaped form in the Taiwanese hotel industry amid the COVID-19 epidemic. Different results are found as the hotel industry is divided between AHBM and ALBM hotel firms. We found that the impact of FF on FP is an inverted U-shaped for the AHBM hotel firms in the lowest and the highest ROE quantiles. This means that FF influences AHBM hotel firms with the worst and best performance and does not influence firms with performance in the 25th, 50th, and 75th quantiles. In addition, the impact of FF on FP follows an inverted U-shaped for the ALBM hotel firms in all ROE quantiles, except the 50th quantile. It implies that FF influences ALBM hotel firms with lower and upper performance and does not influence companies with performance in the median. Overall, the findings lend support that the FF has value in difficult market conditions, especially the COVID-19 epidemic period.

The empirical results of this study elicit several practical implications. First, policymakers should develop FF policies that enable companies to respond positively amid a crisis, such as financial difficulty during an epidemic, and maintain effective investment policies. Second, in the terms of hotel managers, whose operating are either AHBM or ALBM, should put more emphasis on their companies' maintenance of FF. Third, from the perspective of investors, the results can be used as a reference for hotel portfolio evaluation. Analysts or investors can compare the FF of a hotel firm against the proposed thresholds to predict their possible future performance.

This study is not free from limitations. First, despite the ongoing COVID-19 epidemic, data were only examined from 2020 Q1 to 2021 Q2, from the TSE listed hotel firms. Given that there are not many hotel firms traded on the TSE, this paper can only use a small sample size of 20 hotel firms (119 quarterly sample observations). Ongoing research should include longer periods of study. Second, given that the hotel industry could vary in different countries, it would be interesting to see if studies based on data from other countries would find a significant nonlinear FF-FP relationship and how different FF influences FF across countries (or sectors/industries). Lastly, Gozgor et al. (69) show that the geopolitical risks negatively affect the capital investment in tourism, and Gozgor et al. (70) reveal that the higher level of legal system quality and the better protection of property rights promote inbound tourism. Future studies should discuss the role of geopolitical risks and institutional quality to improve our understanding of the COVID-19 effect in the hotel sector.

## Data Availability Statement

The original contributions presented in the study are included in the article/supplementary files, further inquiries can be directed to the corresponding author.

## Author Contributions

XZ, B-GC, and K-SW: conceptualization. B-GC and K-SW: methodology, formal analysis, writing—original draft, and writing—review and editing. B-GC: software and data curation. XZ: visualization. All authors have read and agreed to the published version of the manuscript.

## Funding

This work was funded by Natural Science Foundation of China (Grant No: 72162028).

## Conflict of Interest

The authors declare that the research was conducted in the absence of any commercial or financial relationships that could be construed as a potential conflict of interest.

## Publisher's Note

All claims expressed in this article are solely those of the authors and do not necessarily represent those of their affiliated organizations, or those of the publisher, the editors and the reviewers. Any product that may be evaluated in this article, or claim that may be made by its manufacturer, is not guaranteed or endorsed by the publisher.

## Footnotes

^1^Sakov and Bicke (63) suggest a data-dependent rule for choosing the number of bootstrap replications (i.e., the size of bootstrap sample): *m* = *kn*^2/5^, in which *m* and *n* are the size of the bootstrap sample and original sample, respectively, and *k* is a constant smaller than or equal to the number of variables (Li, Sun, & Zou, 2009). This rule in our case leads to *m* = 55.
